# Enumeration and Characterization of Human Memory T Cells by Enzyme-Linked Immunospot Assays

**DOI:** 10.1155/2013/637649

**Published:** 2013-11-11

**Authors:** Sandra A. Calarota, Fausto Baldanti

**Affiliations:** S. S. Virologia Molecolare, S. C. Microbiologia e Virologia, Fondazione IRCCS Policlinico San Matteo, Via Taramelli 5, 27100 Pavia, Italy

## Abstract

The enzyme-linked immunospot (ELISPOT) assay has advanced into a useful and widely applicable tool for the evaluation of T-cell responses in both humans and animal models of diseases and/or vaccine candidates. Using synthetic peptides (either individually or as overlapping peptide mixtures) or whole antigens, total lymphocyte or isolated T-cell subset responses can be assessed either after short-term stimulation (standard ELISPOT) or after their expansion during a 10-day culture (cultured ELISPOT). Both assays detect different antigen-specific immune responses allowing the analysis of effector memory T cells and central memory T cells. This paper describes the principle of ELISPOT assays and discusses their application in the evaluation of immune correlates of clinical interest with a focus on the vaccine field.

## 1. Introduction

The ELISPOT assay was originally developed to detect antigen-specific antibody-secreting B cells [1] and then evolved for the enumeration of antigen-specific cytokine-secreting T cells [2]. Since then the ELISPOT assay has been used to measure antigen-specific T cells by detecting cytokines secreted by T cells after stimulation with synthetic peptides (used as a pool or as single peptides) or whole antigens (such as proteins or lysates).

The ELISPOT assay not only permits the quantification of T cells that respond to a specific antigen but it also allows the detection of functionally relevant molecules upon specific stimulation of effector T cells. Although the tetramer staining method provides valuable information regarding the frequency of T cells (particularly CD8^+^ T cells), it needs to be combined with intracellular cytokine staining for the functional evaluation of T cells. However, the tetramer staining approach requires knowledge of the relevant epitope(s) and its restricting MHC molecule, limiting the use of this approach to the clinical setting. Thus, the ELISPOT is an attractive alternative because it is not limited by HLA restriction. Additionally, the low number of cells required to accurately assess T-cell activity (roughly tenfold less cell material than flow cytometry-based assays) as well as the high sensitivity and specificity of the assay makes the ELISPOT an optimal method for clinical monitoring [3], especially in clinical settings when the number of patient cells is limited (e.g., immunosuppressed subjects or pediatric patients). However, ELISPOT does not allow phenotypic characterization of antigen-stimulated T cells. Thus, magnetic isolation or depletion of T-cell subsets from peripheral blood mononuclear cells (PBMCs) is required to characterize antigen-specific CD8^+^ or CD4^+^ T-cell responses or both. Studies have shown that the ELISPOT assay provides reproducible results among different laboratories when the assay procedure and data analysis are standardized [4–6].

The interferon-gamma (IFN-*γ*) ELISPOT is the most widespread format used to measure and monitor antigen-specific T-cell responses induced by both prophylactic and therapeutic vaccine candidates. IFN-*γ*, a cytokine with antiviral, immunoregulatory, and antitumor properties [7], is produced by CD4^+^ T helper cell type 1 lymphocytes and CD8^+^ cytotoxic T lymphocytes (CTL) as part of the adaptive immune response. Additional cytokines, such as interleukin (IL)-2, IL-4, IL-5, IL-10, IL-12, IL-17, and tumor necrosis factor-*α*, can be evaluated by the ELISPOT assay, and kits are commercially available. Additionally, key molecules involved in the mechanism of cell-mediated cytotoxicity, such as granzyme B and perforin, have also been evaluated in an ELISPOT platform providing a more comprehensive evaluation of antigen-specific effector CTL functions [8].

A dual ELISPOT assay for the simultaneous detection of two cytokines, such as IFN-*γ* and IL-2, has been established, using a typical enzymatic approach [9] or using a specific fluorophore (FluoroSpot) [10]. An automated analysis of triple-color FluoroSpot for cytokine secretion has also been described [11]. A particular limitation is the availability of automated readers designed for the analysis of spots in three colors.

The goal of an efficient vaccine is to generate long-lived memory T cells capable of recognizing and rapidly expanding in response to infections [12]. Briefly, the three phases involved in the generation of a memory T-cell response are: activation and expansion, contraction, and differentiation into memory. After the initial phase of activation and expansion, the majority of effector T cells die, but the remaining 5–10% persist in the host and further mature into a stable pool of memory T cells. Such primed memory cells are maintained for a prolonged period of time after immunization and studies in humans indicate that memory responses can be maintained for decades [13, 14].

Memory T-cell populations are heterogeneous and consist of two broad categories of T-cell subsets that vary in their homing characteristics as well as their effector and proliferative functions. Expression of the lymph node homing receptors CCR7 and CD62L is the most frequently used to define the memory T-cell subsets. The effector memory T cells are typically CCR7^−^ CD62L^−^ CD45RA^−^ and the central memory T cells are characterized as CCR7^+^ CD62L^+^ CD45RA^−^ [15]. Effector memory T cells traffic through nonlymphoid tissues and secrete mainly effector molecules (IFN-*γ*) and cytolytic molecules (perforin) while exhibiting reduce proliferative capacity. Central memory T cells express lymph node homing receptors and lack immediate effector function but exhibit robust proliferative capacity and elaborate effector molecules upon secondary stimulation.

This paper describes the principle of the ELISPOT assay as well as the types of ELISPOT assays used to enumerate and characterize human memory T cells based on IFN-*γ* production and discusses their application in the evaluation of immune correlates after vaccination.

## 2. The Principle of the ELISPOT Assay

**Figure 1:**
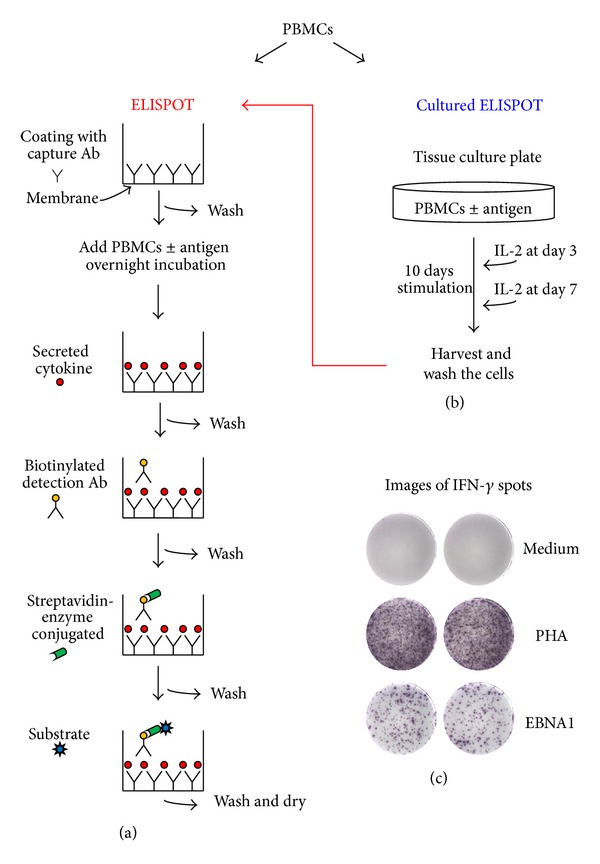
Schematic representation of ELISPOT assays. (a) The standard ELISPOT is used to measure antigen-specific effector T cells. The membrane's plate is coated with an antibody (Ab) specific for the cytokine of interest (capture Ab). Peripheral blood mononuclear cells (PBMCs) are added with or without antigen and incubated overnight. The cytokine released by the cells binds to the capture Ab. After washing, an anti-cytokine biotinylated detection Ab is added followed by streptavidin conjugated with an enzyme, and finally an enzyme substrate is added which produces colored spots. (b) The cultured ELISPOT assay is used to measure antigen-specific memory T cells. PBMCs are cultured with or without antigen for 10 days, with the addition of interleukin-2 (IL-2) at days 3 and 7, allowing the expansion of antigen-specific T cells. Then, cells are restimulated with the same antigen used during the 10-day period in the ELISPOT assay. (c) Spots are counted using an automated ELISPOT reader. Representative images of interferon-*γ* (IFN-*γ*) ELISPOT wells are shown. PBMCs from an Epstein-Barr virus (EBV) seropositive healthy subject were evaluated in response to medium alone (negative control), phytohemagglutinin (PHA, positive control) and a peptide pool (15 amino acids in length with an 11 amino acid overlap) spanning full-length EBV latent protein EBNA1.

The IFN-*γ* ELISPOT assay is carried out in commercially available synthetic membrane-bottomed 96-well plates. Plates are first coated with an IFN-*γ*-specific antibody (capture antibody). Then, PBMCs are added in the presence or absence of antigens and incubated for 18–24 hours. This incubation step allows responding cells to recognize the corresponding antigen and secrete IFN-*γ*, which is captured by the antibody. Cells are removed by washing and a biotinylated detection antibody directed to an epitope on IFN-*γ* distinct from that recognized by the capture antibody is added. Then, streptavidin conjugated with an enzyme (usually alkaline phosphatase or horseradish peroxidase) is added. Finally, a precipitated substrate for the streptavidin-linked enzyme is added, and plates are incubated until spots emerge. Each colored spot represents a single cell producing IFN-*γ*, and spot numbers are counted with an image-based spot reader with the accompanying analysis software. The readout of the assay is the number of IFN-*γ* secreting cells per number of cells plated.  Figure 1(a) illustrates the steps involved in the ELISPOT assay.

Different cytokines have different secretion kinetics, which needs to be taken into account when measuring other cytokines rather than IFN-*γ* [3]. Cytokines such as IL-4, IL-5, or IL-17 require a longer (40 or 48 hours) activation period [16, 17]. While IFN-*γ* is an abundant cytokine, other cytokines (such as IL-2, IL-5, and IL-13) are produced by specific T cells present at a lower frequency [18].

ELISPOT assays are typically performed with total PBMCs, which contain sufficient antigen-presenting cells (APCs) for T-cell stimulation. Furthermore, depletion of specific T-cell subsets allows the characterization of antigen-specific T-cell responses. Commonly, T-cell depletion is performed using anti-CD4^+^ or anti-CD8^+^ antibody-coated immunomagnetic beads before starting the stimulation period with the specific antigens and corresponding controls.

The assay can be performed either with fresh or frozen PBMCs. After thawing, it is recommended to rest the cells before stimulation. In fact, an increase in background when cells are thawed just before plating and stimulation has been observed, while the lowest background levels are achieved by resting the cells from 6 hours to overnight [19]. In our laboratory, PBMCs are rested overnight (16–18 hours) in culture medium under humidified 37°C, 5% CO_2_ conditions. A resting period (at least 1 hour under humidified 37°C, 5% CO_2_ conditions) before stimulation should also be considered when working with freshly isolated PBMCs [19].

The choice of the antigen is critical. When proteins are used as antigens, APCs preferentially process the antigen(s) via the exogenous pathway of antigen presentation leading primarily to presentation of peptides on MHC class II molecules and stimulation of CD4^+^ T cells. However, a preincubation step prior plating the cells in the ELISPOT assay is required for optimal antigen presentation via MHC class II [20]. On the other hand, stimulation of CD8^+^ T cells requires the antigen to be processed via the endogenous pathway of antigen presentation, leading to the presentation of peptides on MHC class I molecules [21]. Peptides can be used as antigens to stimulate CD8^+^ T-cell responses through the direct binding to the MHC molecules avoiding the need for the endogenous pathway of antigen presentation. However, if peptides are of the proper length, they can be used to stimulate both CD8^+^ and CD4^+^ T-cell responses. Most immunologists use an entire protein-spanning mixture of overlapping peptides, presented by a variety of HLA alleles, for *in vitro *stimulation of T lymphocytes, allowing the HLA-unrestricted evaluation of T-cell responses. Sets of peptides, 15 amino acids in length with an 11 amino acid overlap, have been shown to represent a good compromise for stimulating both CD8^+^ and CD4^+^ T-cell responses in a number of applications [22, 23]. They are useful tools for screening and monitoring T-cell responses overtime. However, if a comparison of antigen-specific CD4^+^ versus CD8^+^ T-cell responses is required, shorter peptides (9 to 10 amino acids) are preferable to detect CD8^+^ T-cell responses [22].

Studies have shown that in general, IFN-*γ* spot forming cells are detected on stimulation with ≥0.1 *μ*g/mL of peptide per pool and, when tested individually, a peptide concentration of 2 *μ*g/mL is sufficient for the detection of antigen-specific T-cell responses [24]. In other studies, the final concentration of each peptide in a pool varied from 10 *μ*g/mL [25] to 200 *μ*g/mL [26]. No consensus on the optimal number of peptides that can be used per pool, which varies from 25 [24] to 123 [27], has been reached yet. A recent study suggests that using higher concentration and increased number of peptides per pool, the proliferation of antigen-specific CD8^+^ T cells may be inhibited [28]. In this study, peptide concentrations of 10 *μ*g/mL showed a marked inhibition of proliferation, as compared to concentrations of 1 *μ*g/mL [28]. The same event was observed when using pools containing 20 or 25 peptides with respect to pools containing 10 peptides [28]. Of note, peptides are usually dissolved in dimethyl sulfoxide (DMSO), which is toxic to cells at concentrations >0.5–1.0% in culture medium [24, 28].

Key controls are needed to measure antigen-specific T-cell responses. Negative control routinely consist of cells cultured in the absence of any stimulus, to assess background cytokine production. Stimulation with the polyclonal mitogen phytohemagglutinin (PHA) is the most common positive control for ELISPOT assays. CEF (cytomegalovirus, Epstein-Barr virus, influenza virus) peptide pools have been proposed as positive control as well as for optimizing and standardizing the assay [29]. These pools consist of peptides corresponding to a defined HLA class I-restricted T-cell epitope from each of these three viruses that are recognized by CD8^+^ T cells. There are also commercially available CEF peptide pools based on defined HLA class II-restricted T-cell epitopes.

Some empirical approaches have been proposed for determining an ELISPOT response [4, 30]. According to Dubey et al. [30] the cut-off for a positive response is ≥55 spots/million cells and ≥4-fold over the mean background (negative control). Similar criteria has also been proposed by other laboratories [19], in addition to defining a cut-off for the positive control (PHA, with at least >500 spots/million cells) and the mean background in negative wells (<30 spots/million cells). It should be noted that the positive criteria apply to the ELISPOT procedures and reagents that were used to validate them.

## 3. Types of ELISPOT Assays

Two types of ELISPOT assay, standard and cultured, have been designed to detect two different T-cell subsets. As mentioned before in the standard ELISPOT assay (also known as *ex vivo* ELISPOT assay), cells are incubated with the antigen for 18–24 hours and the cytokines that are released during that time by antigen-specific cells are captured by a monoclonal antibody allowing enumeration. This assay allows the measurement of T cells capable of immediate secretion of IFN-*γ* upon antigen stimulation. These cells are thought to represent mainly effector memory T cells.

Central memory T cells require antigenic restimulation to develop effector function, and the cultured ELISPOT assay is thought to comprise mainly these memory T cells, that is, central memory T cells [31]. The cultured ELISPOT assay requires a period of *in vitro* lymphocyte culture before the standard ELISPOT assay is performed (Figure 1(b)). Currently, we are performing this assay by culturing lymphocytes (5 × 10^5^ cells/mL per well in a 48-well tissue culture plate) with specific antigens for 10 days allowing T cells to expand in response to the antigen. On days 3 and 7, half of the supernatant from each well is removed and replaced with fresh culture medium supplemented with 20 IU/mL recombinant human IL-2. After 10 days, cells from each well are harvested and washed before their use in the ELISPOT assay. Thus, antigen-stimulated cells are added to the ELISPOT plate and restimulated with the same antigen used for stimulation during the 10-day period. Variations in the cultured ELISPOT methodology (summarized in Table 1) include the length of the cultures, the tissue culture plate used, the concentration of IL-2 and the frequency with which it is replenished, and whether or not cultured cells are washed and rested before the performance of the ELISPOT assay. After subtracting from the number of spots in antigen-stimulated wells the number of spots in medium-stimulated wells, the results may be referred to as antigen-specific spot-forming cells per million input PBMCs [32], while others normalize the antigen-specific spots per million PBMCs to the proliferation index (number of antigen-stimulated cells divided by the number of medium-stimulated cells), to take into account the expansion of T cells during the culture [33, 34].

It can be argued that the cultured ELISPOT assay increases the sensitivity of the standard ELISPOT assay by increasing the detection of low frequency antigen-specific T-cell responses [35, 36]. However, several studies have shown that the responses detected by *ex vivo* and cultured ELISPOT assays do not correlate with each other in response to HIV [33], EBV [34], hepatitis C virus [37], and malaria [38] antigens, by comparing the frequency of *ex vivo* response against the frequency of cultured response to the same antigen. These results suggest that different IFN-*γ*-producing cells are being evaluated, reflecting the different nature of these two assays.

It can also be argued that the stimulation protocol might induce *in vitro* priming of naïve T cells. However, we consider that it is unlikely to see significant *de novo* priming since the assay is of short duration (10 days) with no addition of peptide antigens other than the initial set-up, no addition of other exogenous cytokines apart from IL-2, and no enrichment of dendritic cells all of which are necessary for *in vitro* primary responses [39].

Evidence indicates that a different population of T cells, most likely central memory T cells that differentiate into effector T cells during the culture period, are measured by the cultured ELISPOT assay, as compared with the measurement of circulating effector memory T cells that are quantified by the standard ELISPOT. Todryk et al. [40] evaluated the effect of depletion of memory T-cell subsets on standard and cultured ELISPOT responses to recall antigens, such as influenza and purified protein derivative (PPD) of *Mycobacterium tuberculosis*. Results from these studies indicate that the depletion of CCR7^+^ or CD62L^+^ (central memory phenotype markers) cells reduced CD4^+^ and CD8^+^ T-cell responses detected by the standard ELISPOT, but the cultured ELISPOT responses were more dramatically reduced, indicating the predominant role of central memory T cells in the cultured ELISPOT response and also suggesting a role for this T-cell subset in the standard ELISPOT response. Godkin et al. [37] examined the effect of depletion of CCR7^+^ T cells on hepatitis C virus-specific standard and cultured ELISPOT responses. While depletion of CCR7^+^ T cells reduced the cultured ELISPOT response, the standard responses were only marginally affected by CCR7^+^ cell depletion, indicating that CCR7^+^ CD4^+^ T cells contribute substantially to the hepatitis C virus cultured memory cells but not to *ex vivo* responses.

**Figure 2:**
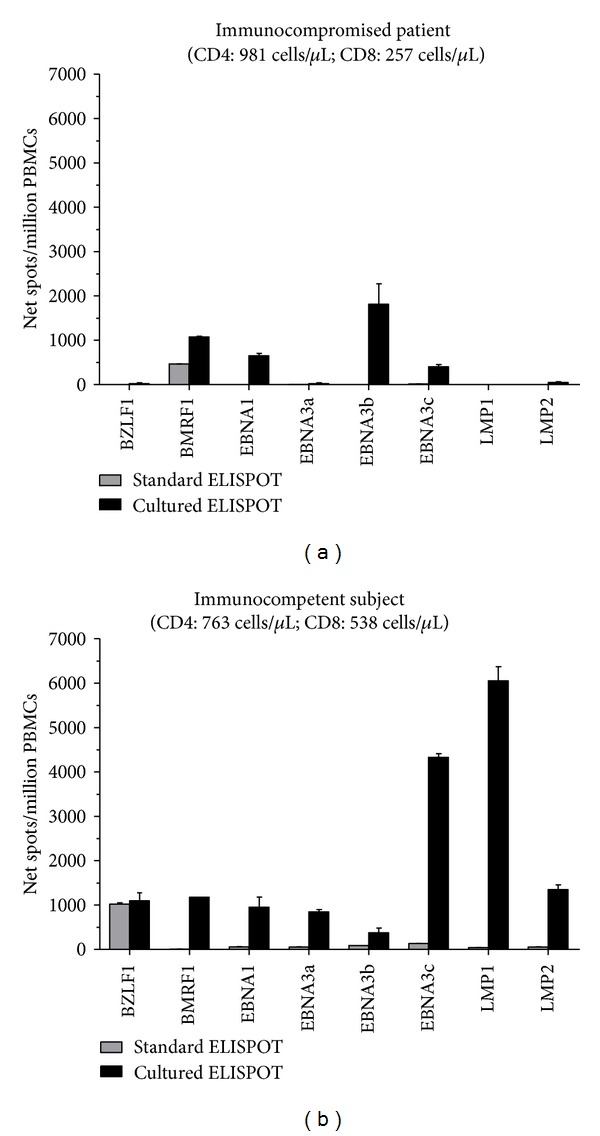
Epstein-Barr virus (EBV)-specific T-cell responses determined by standard and cultured ELISPOT assays. Peripheral blood mononuclear cells (PBMCs) from (a) an EBV seropositive immunocompromised patient (an hematopoietic stem cell transplant recipient suffering from a EBV-associated posttransplant lymphoproliferative disorder) and (b) an EBV seropositive healthy subject were analyzed by standard and cultured ELISPOT assays in response to peptide pools (15 amino acids in length with an 11 amino acid overlap) representing the full-length lytic (BZLF1 and BMRF1) and latent (EBNA1, EBNA3a, EBNA3b, EBNA3c, LMP1, and LMP2) EBV proteins. The mean number of spots from duplicate wells was adjusted to million PBMCs. Results are shown as net spots/million PBMCs calculated by subtracting the mean number of spots in wells with cultured medium only from the mean number of spots in wells from each EBV peptide pool. Results from the cultured ELISPOT were adjusted per proliferation index (number of antigen-stimulated cells after 10 days of culture divided by the number of medium-stimulated cells after 10 days of culture). Error bars represent the standard error of the mean. T-cell subsets indicated are at the time of ELISPOT analyses.

Studies on an HIV-infected patient with residual HIV replication during antiretroviral treatment and with virus variants with several stop codons in the reverse transcriptase gene have shown robust Pol-specific cultured ELISPOT responses [41]. Furthermore, recent studies on elite controllers (HIV-infected individuals that control viral replication to levels below the limit of detection by standard clinical assays in the absence of antiretroviral treatment) indicate that expandable antigen-specific T cells measured by cultured ELISPOT from these individuals predominantly display a central memory phenotype [42]. Additionally, we have shown that standard and cultured ELISPOT responses to HIV [33] and EBV [34] antigens are different not only in frequency but also in the range of antigens recognized.  Figure 2 dissects standard and cultured EBV-specific T-cell responses in an immunocompromised patient (an hematopoietic stem cell transplant recipient suffering from a EBV-associated posttransplant lymphoproliferative disorder) and a healthy subject with remote EBV infection. EBV-specific standard and cultured ELISPOT responses in the EBV seropositive healthy subject differed in both frequency and range of antigens recognized, and only few EBV-specific T-cell responses were detected in the patient with EBV-associated disease, which generally were of low frequency compared to the healthy subject.

## 4. Correlates of Immune Control

A better understanding of correlates of protective immunity against pathogens would be beneficial in designing vaccines. Studies performed in mice, infected with lymphocytic choriomeningitis virus or the intracellular bacterium *Listeria monocytogenes,* have shown that central memory T cells have a greater capacity than effector memory T cells to persist *in vivo* and are more efficient in mediating protective immunity because of their increased proliferative capacity [43]. Studies performed in humans have suggested that a CD4^+^ T-cell response to a conserved epitope in the circumsporozoite protein detected by cultured IFN-*γ* ELISPOT, rather than the *ex vivo* assay, correlated with protection from malaria infection and disease [44]. However, the protective capacity of different memory T-cell subsets may depend on the pathogen in question. Bachmann et al. [45] have reported that to protect mice against vaccinia virus, high numbers of effector memory T cells were required in peripheral tissue before viral challenge. Additionally, blood antigen-specific CD8^+^ effector memory T cells induced by immunizations with adenoviral and modified vaccinia Ankara vectors expressing pre-erythrocytic malaria antigens correlate with protection against malaria liver-stage infection [46].

Studies performed in rhesus macaques, immunized with plasmid DNA and replication defective adenoviral vectors encoding simian immunodeficiency virus (SIV) proteins and then challenged with pathogenic SIV, indicate that the preservation of vaccine induced antigen-specific central memory CD4^+^ T cells is essential for better outcome and survival following pathogenic SIV challenge [47]. Others suggest that central memory CD8^+^ T cells correlate with protection against SIV in rhesus macaques immunized with a vaccine candidate (DNA prime followed by a boost with the highly attenuated NYVAC-based SIV vaccine) [48]. It was found that the level of SIV replication following challenge exposure correlated inversely with the magnitude of vaccine-elicited SIV-specific central memory CD8^+^ T-cell responses but not with CD8^+^ effector memory T-cell responses [48]. However, it has been recently reported that SIV vaccines that include rhesus cytomegalovirus vectors elicited high-frequency SIV-specific effector memory T-cell responses at mucosal surfaces that control highly pathogenic SIV infection after mucosal challenge, suggesting a role for effector memory T cells as correlate of protection [49].

Studies have also shown that antigen-specific T-cell responses measured by cultured cellular assays, but not by standard ELISPOT, correlate with low viremia and high CD4^+^ counts in HIV-1-infected individuals naïve to antiretroviral therapy [33]. Yang et al. [50] investigated if hepatitis B-specific T-cell responses induced by DNA vaccination in 12 chronic hepatitis B carriers treated with lamivudine might be responsible for suppression of viral rebound after stopping the therapy. After discontinuation of the combined therapy, 6 patients showed undetectable viral load (virological responders) and the other 6 patients had high levels of viral load (nonvirological responders). Virological responders had higher antigen-specific *ex vivo* T-cell responses compared to nonvirological responders at the end or just before the discontinuation of the treatment, but only virological responders had detectable cultured ELISPOT responses for at least 40 weeks after the last vaccination. These data suggest that the cultured ELISPOT might be a better predictor of virological response than *ex vivo* ELISPOT [50]. T-cell responses to RD1 (region of difference 1 protein) antigens have been analyzed in patients with either severe or mild active tuberculosis as well as in successfully treated individuals (control subjects) and in subjects with negative tuberculin skin test results (negative control subjects) [51]. These studies have shown that in patients with severe active tuberculosis, both CD4^+^ T cell-mediated effector memory and central memory responses to the selected RD1 peptides were almost absent, while these responses were found in the majority of the patients with mild active tuberculosis. In contrast, recognition of the selected RD1 peptides was detected in the control subjects only by expanding the central memory T-cell pool [51].

Notably, the antigen-specific cultured ELISPOT response induced by a vaccine against malaria (heterologous DNA prime/boost with viral vectors) correlated significantly with the degree of protection against malaria sporozoite challenge. In contrast, antigen-specific standard ELISPOT responses did not predict protection against experimental malaria from sporozoite challenge [32]. This study established the cultured ELISPOT as a correlate of protection in malaria vaccine clinical trials [31].

The major goal in the field of T cell-inducing vaccine development (against, e.g., HIV, malaria, and tuberculosis) is the induction of long-term memory responses. In recent studies, cultured ELISPOT assays have proven to be a valuable tool for studying T-cell memory in HIV-1 vaccine trials [52, 53].

## 5. Conclusions

The ELISPOT is a straightforward, sensitive, specific, and widely utilized method for measuring T-cell responses after natural infection or vaccination. However, the frequency of T-cell responses obtained by the standard ELISPOT, quantifying effector memory T cells, does not correlate well with disease control or protection against challenge with some vaccine candidates. The cultured ELISPOT, on the other hand, quantifying central memory T cells, correlates with slow disease progression in natural infection and immune protection with several experimental vaccines. Thus, the two types of ELISPOT assays provide different information regarding T-cell-mediated immunity.

## Figures and Tables

**Table 1:** Variants of cultured ELISPOT protocols.

Reference	Length of culture	Size of culture plate	Cells per well in culture plate	Concentration and addition of IL-2	Washing and resting cells before ELISPOT
Godkin et al. 2002 [37]	12 days	96-well plate	2 × 10^5^	10 U/mL on days 3, 6, and 9	Washing cells only
Pinder et al. 2004 [38]	14 days	Not described	1 × 10^6^	10 U/mL on days 5 and 10	Washing cells only
Reece et al. 2004 [44]	14 days	48-well plate	1 × 10^6^	10 U/mL on days 5 and 10	Washing cells only
Keating et al. 2005 [32]	10 days	24-well plate	1 × 10^6^	10 U/mL on days 3 and 7	Washing and resting cells overnight
Goonetilleke et al. 2006 [35]	11 to 13 days	Not described	2 × 10^6^	1800 U/mL on days 3 and 7	Washing and resting cells overnight
Calarota et al. 2008 [33]	12 days	24-well plate	5 × 10^5^	10 U/mL on days 3 and 7	Washing cells only
Todryk et al. 2009 [40]	10 days	24-well plate	1 × 10^6^	100 U/mL on days 3 and 7	Washing and resting cells overnight
